# Determination of the optical interference of iron oxide nanoparticles in fluorometric cytotoxicity assays

**DOI:** 10.1016/j.heliyon.2024.e25378

**Published:** 2024-01-29

**Authors:** Leisha Martin, Kimberly Lopez, Shayden Fritz, Charles P. Easterling, Jacob A. Krawchuck, Agus R. Poerwoprajitno, Wei Xu

**Affiliations:** a Department of Life Sciences, College of Science, Texas A&M University - Corpus Christi, Corpus Christi, TX, 78412, USA; b Department of Physical and Environmental Sciences, College of Science, Texas A&M University - Corpus Christi, Corpus Christi, TX, 78412, USA; c Center for Integrated Nanotechnologies, Sandia National Laboratories, Albuquerque, NM, 87185, USA

**Keywords:** Iron oxide nanoparticles (IONPs), A549 cells, Cytotoxicity, Nanoparticle toxicity, Assay, Optical interference

## Abstract

Nanomaterials are known to exhibit unique interactions with light. Iron oxide nanoparticles (IONPs), composed of magnetite (black iron oxide) specifically, are known to be highly absorptive throughout the visible portion of the spectrum. We sought to investigate and overcome optical interference of IONPs in colorimetric, fluorometric and luminescence assays by introducing additional controls and determining the concentration-dependent contribution to optical artifacts which could confound, skew, or invalidate results. We tested the in vitro cytotoxicity of ∼8 nm spherical magnetite nanoparticles capped with alginate on a human lung carcinoma (A549) cell line for different exposure periods and at various concentrations. We observed significant interference with both the MTT reagent and the absorption at 590 nm, a concentration-dependent reduction in the luminescence, fluorescence at ∼490 nm (viability marker), and fluorescence at 530 nm (cytotoxicity marker). After introducing an additional correction, we obtained more accurate results, including a clear decrease in viability at 12-h post-treatment, with apparent near complete recovery after 24-h in addition to a dose-independent, time-dependent alteration in the cell proliferation rate. A small increase in cytotoxicity was noted at the 24-h timepoint at the two highest concentrations. According to our results, the MTT reagents appear to interact substantially with IONPs at concentrations above 0.1 mg/mL, therefore, this assay is not recommended for IONP cytotoxicity assessment at higher concentrations.

## Graphical abstract

**Figure:**
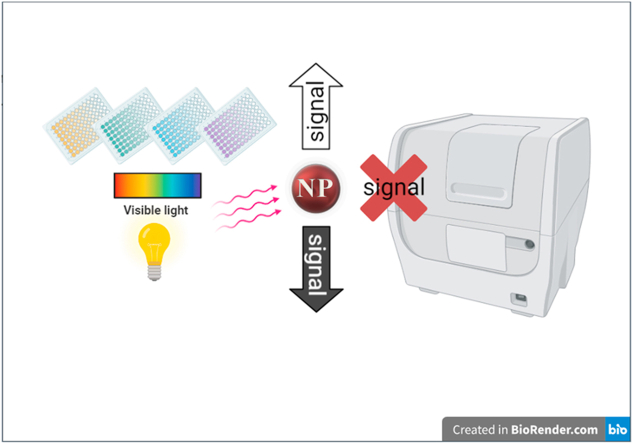

## 1 Introduction

Iron oxide nanoparticles (IONPs) are among the most essential nanomaterials (Seabra and Haddad 2014 [1], due to their abundant prospective applications in a range of biomedical, general scientific, and engineering disciplines. IONPs are of interest for drug or other therapeutic agent delivery [2], magnetic resonance imaging (MRI) contrast agents [3], cell-tracking [4], and magnetic hyperthermia/thermotherapy [5,6]. In addition, other applications have been proposed, not are by no means limited to magnetic data storage and recording [7], catalysts [8], water remediation [9] and energy storage applications [10,11] with new applications frequently being proposed. Of particular interest are their unique size-tunable magnetic and electronic properties. It is possible that the characteristics of these materials, which make them so attractive, also prove to be detrimental when consideration of a biological system is taken [12].

Animal models have revealed a link between inhaled particles and murine lung inflammation [13] and lung cancer [14,15]. Although the dextran-coated IONP solution, finding application as the IV-administered MRI contrast agent Feridex®, has received FDA approval for human use in the United States; there still exists a significant knowledge gap regarding exposure to NPs in general, but more specifically, the effects of IONPs on cell viability, proliferation, and normal cell processes [16]. In fact, many researchers have reported that the use of these particles can exert severely detrimental actions on the living cell [16]. Some negative observations include lactate dehydrogenase (LDH) leakage and elevated concentrations of proinflammatory factors [17], significant reductions in viability in murine and human cells [[18], [19], [20]], and decreased cell proliferation [18,21] and migration [21].

Nanoparticles, known to have unique optical properties, can interact with a range of electromagnetic wavelengths and even minuscule changes in size are known to dramatically shift their emission spectra [[22], [23], [24]]. These materials have been known to, “hoax” researchers in viability assays for nearly two decades [25]. Kroll and co-workers published a thorough research study on 24 nanomaterials investigating several mechanisms by which engineered nanoparticles may alter the results of common cytotoxicity assays [26]. Numerous researchers have reported optical interference of nanomaterials in traditional assays, proposed alterations to existing protocols, introduce additional controls, and have even gone so far as to question the feasibility of using these assays for nanoparticle cytotoxicity studies [[27], [28], [29], [30], [31], [32], [33], [34], [35]]. Despite what appears to be some increasing awareness regarding this issue, classic assays are still commonly used in nanoparticle toxicitvy studies without the incorporation of appropriate controls.

Although a range of nanomaterials have been discussed in the literature, being non-fluorescent, highly absorptive semiconductors, IONPs have specific optical properties to consider. We previously observed optical interference in a fluorometric assay when used in combination with IONPs, which we attributed to the high optical absorption of magnetite, or black iron oxide [36]. To address this issue, we have incorporated several controls to identify and compute the interference (in the form of signal reduction, wavelength shifting, and or signal enhancement) due to IONPs in combination with the light and/or the assay reagents. The results of our study may explain some inconsistencies in toxicity assessment results in published studies that have employed these assays. The IONP toxicity data measured in different assays provide a reference for the proper use of cellular assays for toxicity assessment with nanoparticles.

## 2 Experimental procedures

### 2.1 Materials and reagents

Acetone, hexane, and ethanol (solvent grade) and were purchased from Fisher Chemical. Alginic acid sodium salt (low viscosity), Fe(acac)_3_, and phenyl ether were purchased from Sigma-Aldrich (St Louis, MO). 1,2-dexadecanediol was purchased from TCI America (Portland, OR). No chemicals were further purified but were used as received from the manufacturer. The ApoTox-Glo™ triplex assay (Catalog No. G6320) was purchased from Promega® (Madison, WI). Staurosporine was purchased from AAT Bioquest, Inc., (Pleasanton, CA) A549, human alveolar epithelial carcinoma cells (ATCC® No. CLL-185) were a gift from Dr. Jay Hong at Northwestern University. Gibco™ Trypsin 2.5 % (Cat. No. 15090046); Dulbecco's Modification of Eagles Medium (DMEM) containing 4.5 g/L glucose, l-glutamine & sodium pyruvate (Corning™ 10-013-CVR, Corning NY); and Cytiva Hyclone™ (SV30010) 10,000 unit/mL penicillin and streptomycin were purchased from ThermoFisher Scientific (Hampton, NH). Avantor® Seradigm fetal bovine serum (FBS) (Product No. 97068-085) was purchased from Avantor via VWR (Radnor, PA).

### 2.2 Magnetite nanoparticle synthesis and polymer capping

**Fig. 1:**
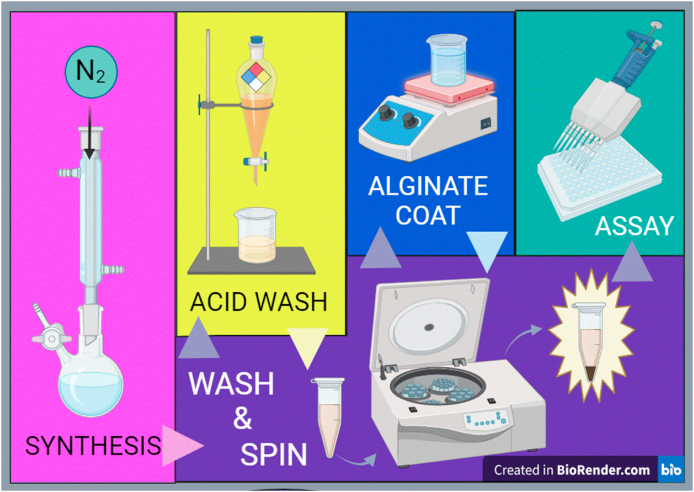
Schematic of IONP synthesis and functionalization procedure used in this study.

The ultrasmall IONP synthesis procedure was performed according to the highly cited method from Ref. [37]. In a typical procedure, phenyl ether (20 mL), is combined with 1,2-hexadecanediol (10 mmol), oleic acid (6 mmol), Fe(acac)_3_ (2 mmol), and oleylamine (6 mmol) in a three-neck, round-bottom reaction flask, under stirring with a condenser and gas adapter, under nitrogen flow and then refluxed for 30 min. After the reflux, the solution became black in color, at which point it was cooled to room temperature and then precipitated and washed with ethanol. The IONPs were washed in a pH ∼1 solution of hydrochloric acid and ethanol and then rinsed 2–3 times with ethanol to remove the organics on the surface. After returning to neutral pH, a solution of 0.2 g IONPs per 20 mL of Milli-Q water was combined with alginate solution with a concentration of 1 g per liter and stirred at 40 °C for 24 h. The suspension was then centrifuged for 3 h at 3000 rpm to collect the alginate-coated particles. The synthesis and functionalization procedure are summarized in Fig. 1.

### 2.3 Structural characterization of magnetite NPs: TEM & XRD

Transmission electron microscope (TEM) images were obtained using a several different instruments for thorough examination: 1.) The FEI Tecnai G2 F20 ST FE-TEM-Materials and the image is presented in Fig. 3 b., 2.) the JEOL 1200 EX TEM operating at 100 kV using a tungsten filament and bottom-mounted (3k x 3k) slow-scan lens-coupled CCD camera (SIA15C) this image is shown in Fig. 3 c., and 3.) the FEI Titan ETEM equipped with Image Ϲs corrector operating at 300 keV shown in Fig. 3 d. & e. Samples were prepared for TEM imaging by depositing a drop of the low-concentration colloidal suspension onto a 200-mesh carbon-coated copper grid. After that, and the solvent dried in air and the sample (IONPs) were fixed onto the grid.

Small Angle X-ray Scattering (SAXS) was performed on a Rigaku SmartLab diffractometer equipped with a HyPix 3000 detector using a Cu–K α source (40 kV and 44 mA) operating at 1.542 Å. SAXS data were fit to a spherical model with a a Gaussian size distribution using Nanosolver v. 3.5 software package (Rigaku). For XRD crystallography, the synthesized IONPs were placed in the sample holder of 2-circle goniometers in a radiation safety enclosure. The 2-circle 218 mm diameter θ - θ goniometer was controlled by the computer and uses stepper motors and optical encoders for the circle having the least angular step size of 0.0001. A 1 kW Cu X-ray tube operating at 40 kV and 25 mA was used as the X-ray source. Bragg-Brentano para-focusing mode was used for X-ray optics with the X-ray diverging from a DS slit (1 mm) at the tube to hit the sample and then to converge at the detector (Lynx-Eye, Bruker-AXS). We use a windows-based software suite to collect and evaluate data. Data is collected by an automated COMMANDER program which employs a DQL file. The program EVA is used to analyze the data. We use a CuKα source (energy is 8.04 keV), corresponding to a wavelength (λ) of 1.5406 Å. The anti-scatter slit was 12.530 mm, and the divergence slit was 1.00 mm. The knife edge was used for anti-air-scatter, and the scan type completed was the coupled theta. The goniometer radius was 217.5 mm. The step size was 0.015, with a start of 5.0 and an end of 70.0.

### 2.4 Nanoparticle characterization: DLS, zeta-potential, and TGA

Small aliquots of alginate-coated IONPs were taken and diluted with filtered Milli-Q water (0.2-μm filter). The samples were placed in a cuvette and analyzed using the Malvern Panalytical Zetasizer Nano. The samples were measured three times to ensure accuracy. Small aliquots of alginate-coated IONPs were taken and diluted with deionized (DI) water or filtered phosphate buffered saline (PBS) (0.2-μm filter). The samples were then placed in a cuvette and placed in Zetasizer. We measured the zeta potential three times to ensure correct measurements.

Thermogravimetric analysis (TGA) was executed on a simultaneous TGA/DSC instrument Netzsch STA 449 Jupiter J3. Samples (5–20 mg) were heated in Al_2_O_3_ crucibles from 20 °C–800 °C at 15 °C/min under air flow (100 mL/min). The data was analyzed using Proteus 8.0 package.

### 2.5 Nanoparticle absorbance measurement

To account for possible interference with the assay, absorbance measurements were performed on the BioTek Cytation 5. 0.1 mg/mL, 0.5 mg/mL, and 1 mg/mL concentrations of colloidal IONPs in DMEM and in water were run at a range of wavelengths from 350 to 850 nm. We also ran blanks at these concentration curves against the fluorescent wavelengths of interest (492 nm, blue and 520 nm, green).

### 2.6 Human lung carcinoma cell growth

**Fig. 2:**
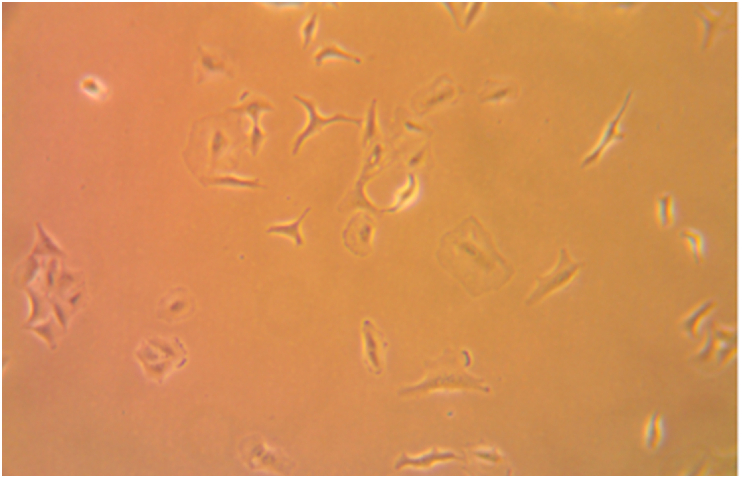
Bright-field image of A549 cells.

Cells were stored in liquid nitrogen in a cryostat until their use. To initiate growth, the sample was thawed and centrifuged, and the culture media was decanted off and disposed. Cells were plated in new a sterile tissue culture flasks with DMEM containing 10 % heat-inactivated FBS, and penicillin-streptomycin. The cells were cultured at 37.0 °C under 5 % carbon dioxide weighted with HEPA-filtered air. After reaching confluence, they were washed with PBS (2x) and removed with 0.05 % trypsin for plating onto 96-well plates. An image of low-density A549 cells provided for reference (Fig. 2).

**Fig. 3:**
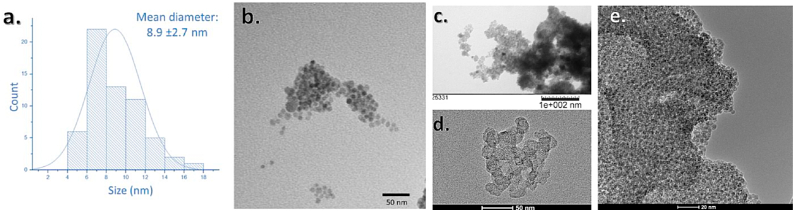
a. Size-distribution histogram from TEM images, **b.** TEM image *scale bar is* 50 nm, **c.** TEM image *scale bar is* 100 nm, **d.** high-resolution TEM image *scale bar is* 50 nm, **e.** broad-field TEM image *scale bar is* 20 nm*.*

### 2.7 Cytotoxicity assay

Bis-alanylalanyl-phenylalanyl-rhodamine 110 (bis-AAF-R110) is a cell-impermeant, fluorogenic peptide marker for dead-cell protease activity. It measures protease enzyme that has been released from lysed cells after they have suffered a loss in membrane integrity. No signal from this marker can generated from viable (intact) cells due to the cell-impermeant property of the peptide; it cannot cross the intact cell membrane. However, dead cells release protease enzymes. These enzymes cleave rhodamine 110 (R110) from the bis-AAF resulting in the fluorescent signal emission. The excitation and emission peaks for R110 are 498 nm and 520 nm, respectively.

Cells were seeded at a concentration of 10,000 cells per well in 96-well plates and a total volume of 100 μL. Media control wells were also included. 0.1, 0.5, and 1 mg/mL of IONPs were prepared in DMEM and incubated with the cells for 6-, 12-, or 24-h exposure time. After the 6-, 12-, or 24-h period, 20 μL of the viability and cytotoxicity reagents (GF-AFC and bis-AAF-R110) were added to the wells simultaneously. Immediately after that, the solutions were mixed on the orbital shaker at 400 rpm for ∼30 s. Then, the plate was then incubated for 45 min at 37 °C before being measured with the BioTek Cytation 5 with an excitation wavelength of 488. Finally, the samples were exposed to 488 nm light for excitation, and fluorescence measurements were taken at 530 nm. The background readings from the wells containing no cells were averaged and subtracted from the obtained averaged readings.

### 2.8 Viability assay

Unlike bis-AAF-R110, glycylphenylalanyl-aminofluorocoumarin (GF-AFC) is a cell-permeant peptide that is used as a fluorogenic marker for live cells. Since live-cell proteases must be detected from within the living cell; having an intact membrane, this substrate must enter the cell by crossing the membrane. Once inside, protease enzymes cleave the AFC from the substrate, triggering the fluorescence signal. The excitation and emission peaks of AFC are 370 nm and 490 nm, respectively. Should the membrane rupture while the substrate is inside the cell, the fluorescence is quenched, and the signal ceases. Therefore, this substrate returns an accurate measure of viable cells.

Experimental wells were seeded with 10,000 cells per well as previously described. Blank wells were filled with 100 μL of DMEM. 0.1, 0.5, or 1 mg/mL of colloidal IONPs were added to experimental wells, and the plates were incubated for 6-, 12-, or 24-h exposure time. Additional controls were used to check for any interference of IONPs with the light and/or the assay. The experimental wells were run in two sets of triplicates. Additional triplicates of each concentration at each time point were measured with IONPs added to untreated cells just before the plate was run on the reader. The actual value of these wells should be identical to the untreated wells and in doing this, signal absorption or interference by the IONPs can be determined. The background readings from the wells containing no cells were averaged and subtracted from the obtained averaged readings. After the 6-, 12-, or 24-h period, 20 μL of the viability and cytotoxicity reagents (both GF-AFC and bis-AAF-R110) were added to the wells simultaneously. Immediately after that, the solutions were moved to the orbital shaker and held at 400 rpm for ∼30 s. After that, the plates were covered in foil and was incubated for 30 min at 37 °C. Finally, foil was removed and plates underwent exposure to 400 nm light for excitation, fluorescence measurements were taken at 492 nm. Measurements were taken with a BioTek Cytation 5 Microplate Reader.

### 2.9 Apoptosis assay

In this assay, cell apoptosis was measured by detecting the apoptosis biomarkers caspase 3 and caspase 7. The luminogenic caspase-3/7 substrate is comprised of a tetrapeptide sequence DEVD (Asp-Glu-Val-Asp), in a reagent optimized for caspase and luciferase activity, thus, for cell lysis (Caspase-Glo® 3/7 Reagent, Promega®). The fluorophore in this assay is luciferase (aminoluciferin), a natural luminescent molecule borrowed from the firefly [38]. A luminescent signal is observed when, upon cell lysis, caspase cleaves the substrate. The intensity of the luminescence is relative to the quantity of caspase activity, and caspase activity gives a direct measure of apoptosis.

In growth medium, 0.1, 0.5, and 1 mg/mL concentrations of IONPs was incubated with the cultured cells for 6, 12, or 24-h exposure time. Staurosporine is known to trigger apoptosis in this cell line and was used as a positive control in this test. 10 μM of staurosporine was applied to the positive control wells. Each well was seeded with 10,000 A549 cells dispersed in growth media, and filled to 100 μL with growth media, as described before. Cells were grown for the respective time periods, as before. The background readings from the wells containing no cells were averaged and subtracted from the obtained averaged readings. After the 6-, 12-, or 24-h period, 100 μL of the Caspase-Glo® 3/7 Reagent was introduced into each well and briefly combined on the orbital shaker (400 rpm for ∼30 s). As an additional control, IONPs in the concentrations of interest were added to positive control wells after measuring the luminescence to determine signal reduction by IONPs. Luminescence measurements were taken on the BioTek Cytation 5 plate reader.

### 2.10 MTT metabolic assay

20,000 cells per well were seeded in 96-well plates with DMEM. Wells were filled to 100 μL/well, as before. Treatments of 0.1, 0.5, or 1 mg/mL of IONPs diluted in growth media were applied to the experimental wells. Plates were incubated for 6-, 12-, and 24 h. The Abcam® MTT kit protocol was followed for the assay. In short, the media was removed from the wells using vacuum aspiration, then 50 μL of MTT solution and 50 μL of serum-free media was added to each well. Then, plates were incubated for 3 h, after which 150 μL of MTT solvent was introduced to each well and the foil-covered plate was moved to the orbital shaker for 15 min. We then measured the absorbance at 590 nm on the BioTek Cytation 5 plate reader. Additional controls consisting of untreated cells, IONPs in water, IONPs in DMEM, and IONPs in DMEM with the MTT reagents added to the wells were also prepared and measured.

### 2.11 Mathematical, statistical, and graphical analyses

TEM image analysis was performed using ImageJ (National Institutes of Health). A size distribution histogram was produced using OriginLAB® (Northampton, Massachusetts). The median values of the assay results, standard deviations, determination of signal reduction, and percentage viability were calculated in Microsoft Excel®. The determination of signal reduction and percent viability was calculated in Microsoft Excel. Statistical outliers were identified and removed from the dataset using GraphPad Prism(R) (Boston, Massachusetts). For signal reduction calculations, an ordinary one-way ANOVA was performed for comparison of the mean values for each test. A two-way analysis of variance (ANOVA) was performed for grouped values for analyzing trends over time and to compare experimental wells to controls, concentration-matched controls to treated wells, concentration-matched wells to untreated wells, and concentration-matched controls to positive controls. All statistical analyses were run in GraphPad Prism®. Values of p < 0.5 (95 % confidence interval) were considered significant, p < 0.01 (99 % confidence interval) were considered very significant, and values of p < 0.001 (99.9 % confidence interval) were considered extremely significant. Graphs of signal reduction in untreated wells were produced using Microsoft Excel® and the slope of the line and percentage reduction were calculated.

## 3 Results

### 3.1 Nanoparticle structural characterization

**Fig. 4:**
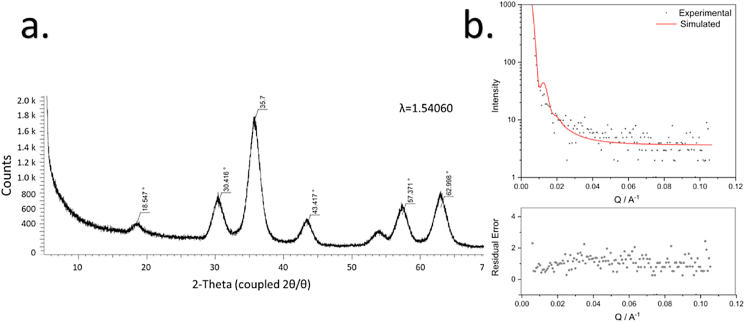
a. XRD spectrum for IONPs reveal magnetite composition and **b.** SAXS data.

TEM imaging was performed for primary size and morphology determination. The size distribution histogram based on imaging results TEM is shown in Fig. 3 a. The TEM images obtained on the various instrumentation described in 2.3 are provided in Fig. 3b-e. The NPs have a spherical morphology and are relatively monodispersed. These particles have a mean diameter of 8.9 nm (±2.7 nm). XRD analysis reveals identical results to those reported by Ref. [37], the black nano-assemblies present with inverse spinel structure and appear to be composed of magnetite (Fe_3_O_4_). The prominent peaks arise at approximately 18.5°, 30.4°, 35.7°, 43.4°, 57.4°, and 63.0°, are attributed to the inverse spinel cubic phase and correspond to the Miller indices: [111], [220], [311], [400], [511], and [440], respectively (Fig. 4a). The SAXS data indicates a particle diameter of 90 nm (RSD = 11 %) when fitted to a and verifies the agglomerate peak (85–90 nm), Fig. 4 b. Agglomeration was also revealed by DLS.

### 3.2 DLS and TGA nanoparticle characterization

**Fig. 5:**
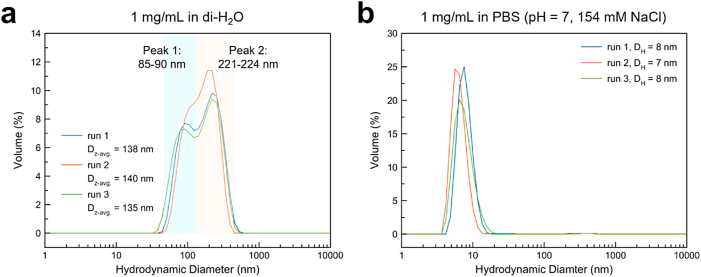
Dynamic light scattering traces of alginate-coated IONPs: a. 1 mg/mL in di-water, b. 1 mg/mL in PBS.

DLS results are provided Fig. 5. The colloidal NPs demonstrate some aggregation in DI water which is apparent by the broad, multimodal distribution in particle size (Fig. 5 a.). The average hydrodynamic diameter given by the first peak is 85–90 nm (range 33–122 nm) and the one given by the second is 221–224 nm (range 125–530 nm). Nanoparticle agglomeration is typically attributed to entropic bonding. These NPs are coated with alginate, a copolymer with two hydroxyl and one carboxyl groups per monomeric unit and is known to form inter- and intramolecular hydrogen bonds [[39], [40], [41]]. Although some of these functional groups will facilitate bonding to the IONP, many will remain unbound and are likely to hydrogen bond with water and with one-another in aqueous solutions, resulting in interparticle hydrogen bonding and agglomeration. However, alginate coated IONPs suspended in PBS (pH = 7, 154 mM NaCl) resulted in colloidally stable single particles with hydrodynamic diameter of 7–8 nm (Fig. 5 b.), which agrees well with single particle size as determined by TEM analysis (D = 8.87 nm). Phosphate, as well as sodium and chloride ions, may serve as counterions to screen electrostatic interactions between the free functional groups on surface-bound alginate, resulting in reduced particle-particle interactions as saline is known to stabilize alginate hydrogels [42].

**Fig. 6:**
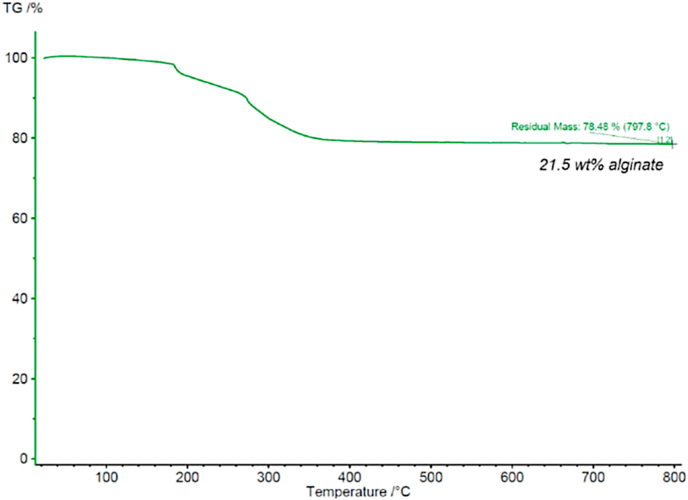
Thermogravimetric analysis of alginate-coated IONPs.

Thermogravimetric analysis (TGA) was performed to quantify the amount of alginate coating on the surface of the IONPs. The IONP sample was heated to 800 °C in air to decompose surface-bound alginate. The TGA curve is shown in Fig. 6 and results reveal a surface functionalization of ∼22 wt% alginate.

### 3.3 Absorbance measurements

**Fig. 7:**
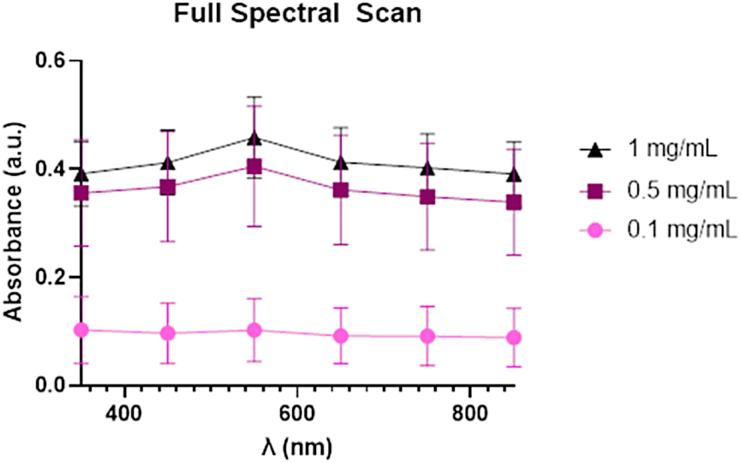
Full absorbance spectrum of alginate-coated, magnetite nanoparticles.

We measured absorbance on the Cytation 5 scanning from 350 to 850 nm and can observe a concentration-dependent increase in absorption throughout this region (Fig. 7). Our previously reported absorbance results for polyethylene glycol (PEG) coated iron oxide (magnetite) NPs showed maximum absorption in the UV region of the spectrum, declining sharply through ^∼^300 nm before leveling off, but remaining above zero through the NIR portion of the spectrum [36], in agreement with the findings of others [[43], [44], [45]]; and [46]. It appears that the particles may reduce the emission signal of the blue and green, fluorescent emitters used in the assay. Although the absorption appears to peak around 525 nm at concentrations ≥0.5 mg/mL, it is clear by the sustained absorption throughout the visible, that the material will enhance the absorption signal of the MTT in the metabolic assay which is run at 590 nm.

### 3.4 Cytotoxicity assay

**Fig. 8:**
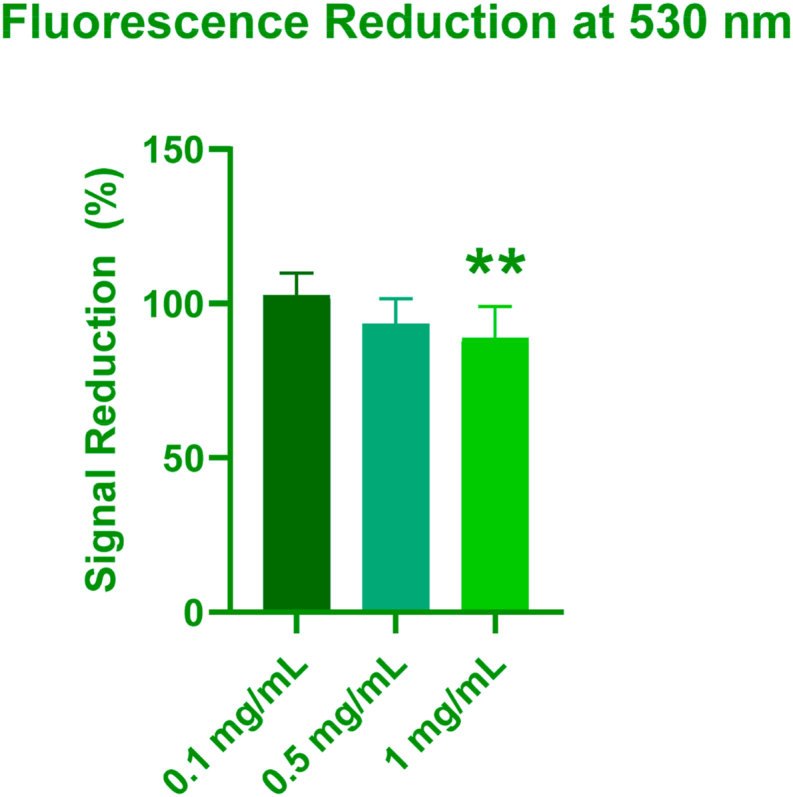
Concentration dependent fluorescence reduction at 530 nm.

We initially compared untreated cells with the measured values for IONPs at the concentrations of interest and the additional control in which IONPs were added to wells containing untreated cells just prior to the measurement. In this case, we would expect the true values to be identical to those of the untreated cells, therefore, optical interference can be calculated. The data is presented in Fig. 8. There was no reduction at the 0.1 mg/mL concentration (n = 9), the average signal reduction at 0.5 mg/mL was 6.5 % (n = 9, ± = 8.0 %), and the average signal reduction for the 1 mg/mL concentration was 11.12 % (n = 9, ± = 10.19 %). There was no statistically significant difference between the untreated (control) cells and the 0.1 mg/mL, or 0.5 mg/mL test wells. The 1 mg/mL signal was found to be statistically significant (p = 0.0055). The correction factor determined was applied to the measured values using the formula.

$true\;signal=\frac{measured\;signal}{0.93492}$, for the 0.5 mg/mL concentration and:

$true\;signal=\frac{measured\;signal}{0.88875}$ , for the 1 mg/mL concentration.

**Fig. 9:**
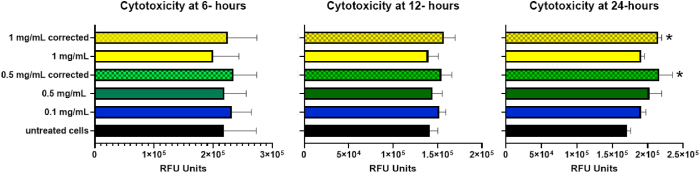
Cytotoxicity (dead cell marker) in A549 cells post-exposure at various timepoints: 6-h post exposure (*left*), 12-h post-exposure (*middle*), and 24-h post-exposure (*right*) *0.01< p < 0.05 when compared to untreated cells.

The calculated values were plotted along with the measured cytotoxicity data for comparison in Fig. 9. The two-way ANOVA multiple comparisons test did not reveal a statistically significant difference between untreated cells and any treatment groups at 6- or 12-h post-treatment. At 24 h, the 0.5 mg/mL corrected value differed slightly from untreated cells (p = 0.0245), whereas the uncorrected, measured value did not. In this case, the correction resulted in statistical significance. The 1 mg/mL corrected value also reached statistical significance vs. the untreated cells (p = 0.0364) while the measured value was not statistically significant. There appears to be a modest, time-dependent, dose-dependent cytotoxic effect that might have been missed, had the correction not been determined and applied.

### 3.5 Viability assay

**Fig. 10:**
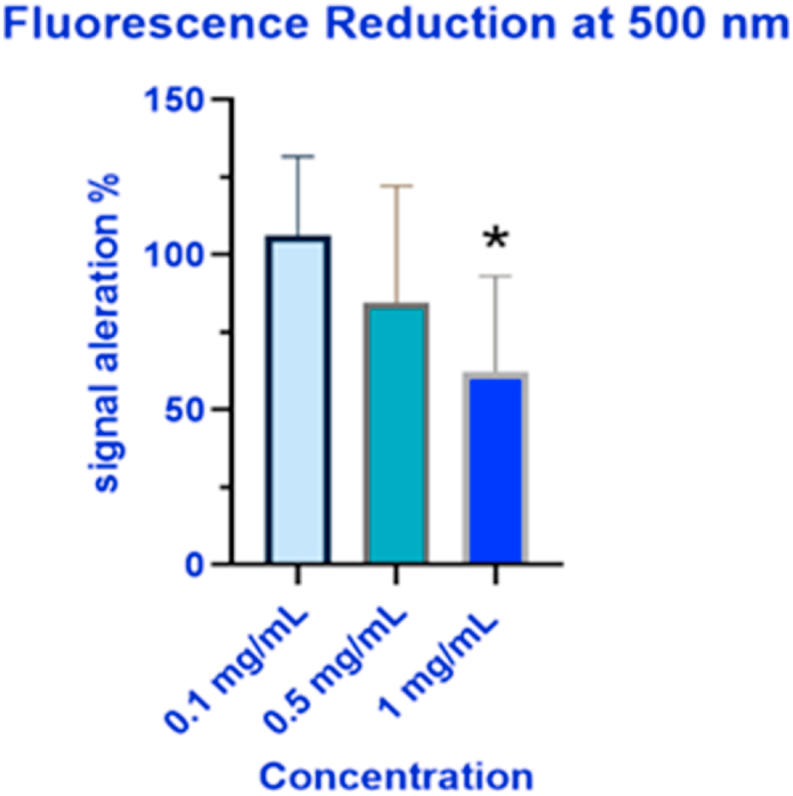
Signal reduction by IONPs at 500 nm.

**Fig. 11:**
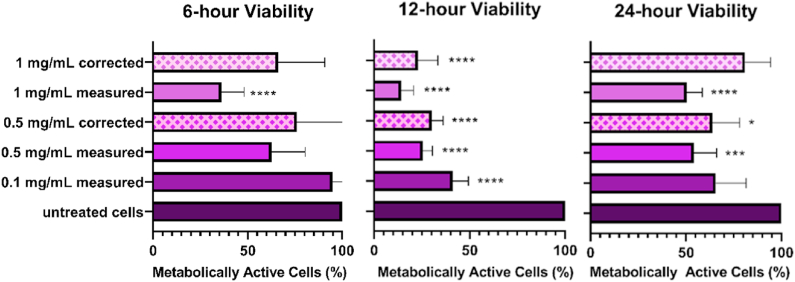
Cell viability in A549 cells after exposure for 6-h (*left*) 12-h (*middle*), or 24-h (*right*) to 0.1, 0.5, or 1 mg/mL concentrations of IONPs.

We gathered the data from the interference tests, (three sets of three) and compared them to the emission signal detected from untreated cells. We observed a concentration-dependent reduction in the viability fluorescence signal which we attribute to light absorption by the IONPs. The signal reduction findings are summarized in Fig. 10. We also calculated the percent signal alteration for each concentration which was found to not significant for the 0.1 mg/mL concentration, 15.44 % (±37.65 %, not significant) for the 0.5 mg/mL concentration, and 37.87 % (±30.93 %) for the 1 mg/mL concentration. The values were plotted on the results chart as correction factors along with measured values and are presented in Fig. 11. We observed statistically significant reductions in viability when comparing the untreated to treated cells at 1 mg/mL (p < 0.0001) which was not met when the correction was applied. We also observed statistically significant reductions in viability at 0.1, 0.5, and 1 mg/mL (p < 0.0001 for all measured and corrected values) at 12 h; and 0.5 mg/mL (corrected p = 0.0108; measured p = 0.002), and the measured value for 1 mg/mL (p < 0.0001), concentrations of IONPs at 24-h. Statistical significance was not met for the 1 mg/mL concentration when the correction was applied.

A statistically significant, concentration-dependent decrease in viable cells was evident at the 12-h time point for all cell types investigated, but by the time the 24-h measurement was taken, the magnetite NP-treated cells appear to have almost completely recovered, and the viable-cell count was in the range of the untreated cells. We observed this exact trend when we investigated PEG-coated magnetite NPs in this cell line [36] and believe it to be a consistent effect. Taking into consideration the range of measured values it appears that the IONPs may not have had observable lethal effects, but simply inhibited cell differentiation over the 6-to-12-h time points as we do not observe a corresponding increase in the dead cell marker at 12 h. In both cases, the growth rate of the NP-treated cells seems to have recovered by 24 h.

### 3.6 Caspase 3/7 level (apoptosis) assay

**Fig. 12:**
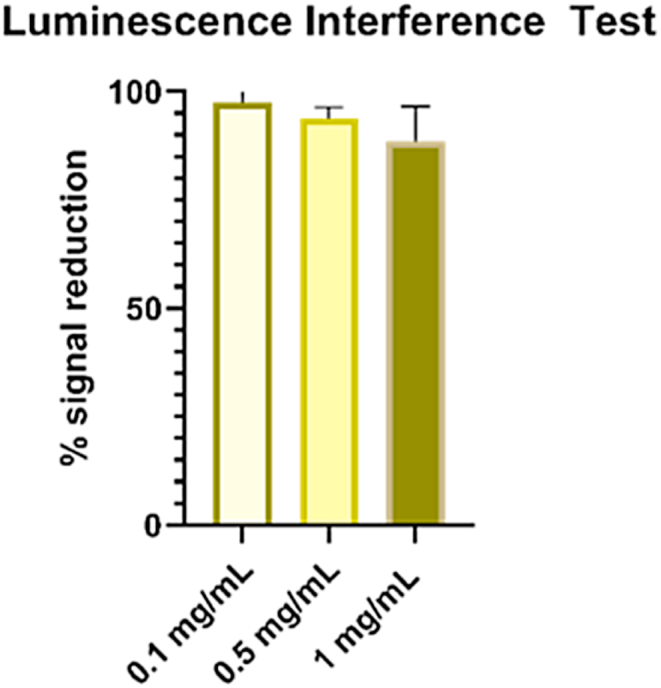
Concentration-dependent reduction in luminescence signal by IONPs.

**Fig. 13:**
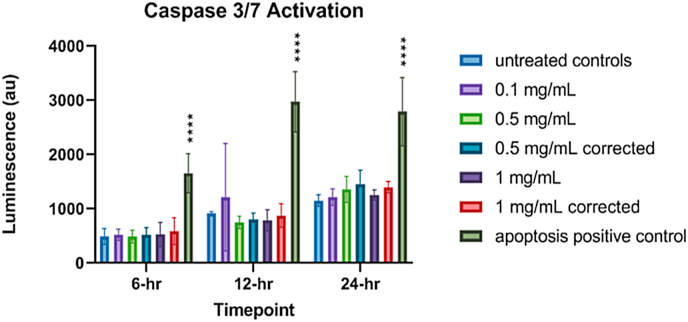
Caspase 3–7 levels in A549 cells after exposure to IONPs at 6, 12, or 24-h ***p < 0.0001 with respect to untreated cells. The apoptosis positive control staurosporine demonstrated statistically significant results, as expected.

Caspases trigger caspase-dependent cell death and are a measure of apoptosis, a mechanism of programmed cell death. Previous studies have found that caspase levels and apoptosis can be triggered by NPs [47,48]. We sought to determine whether IONP treatment increases caspase levels in the cells. However, first, it was necessary to determine whether the IONPs would interfere with or reduce the signal from the luminescence marker. The results of the luminescence interference test showed a small, concentration-dependent signal reduction averaging 2.45 % (±4.53 %) at 0.1 mg/mL, 6.92 % (±2.53 %) 0.5 mg/mL and an 11.53 % signal reduction at 1 mg/mL (±8.093 %). These findings are shown in Fig. 12. None of these.

signal reductions were found to be statistically significant, however, the correction factor was still applied to the 0.5 mg/mL and the 1 mg/mL concentrations and is plotted on the results chart in Fig. 13. None of the treated cells had caspase.

levels significantly higher than the controls. The wells treated with the positive control, staurosporine, produced a statistically significant deviation from control at all time points (p < 0.0001) and therefore had the highest caspase levels, as expected. No statistically significant deviation from control was noted in the NP-treated cells, including the measured or corrected values at any time point investigated. Therefore, it is unlikely that IONPs trigger apoptosis via caspase activation as a mechanism of toxicity.

### 3.7 MTT assay

**Fig. 14:**
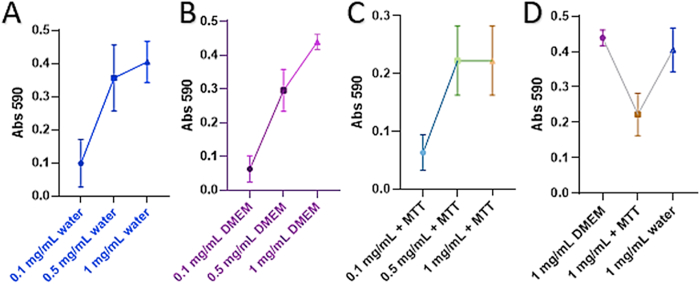
IONP measured at 590 nm in water (A), DMEM (B), DMEM with MTT reagent (C), and comparison of abs 590 values of 1 mg/mL IONPs in DMEM, water and with MTT (D).

**Fig. 15:**
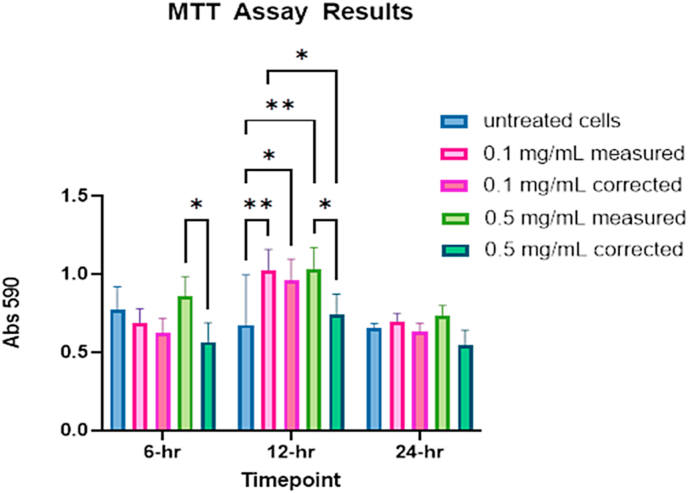
Results of MTT assay for IONPs at various timepoints *p ≤ 0.05, **p ≤ 0.01.

In comparing the IONPs absorption curves at 590 in water, DMEM, and DMEM with MTT, IONPs absorb light strongly as demonstrated in Fig. 14A-C. They also appear to interact with the MTT reagent at, and probably above, 1 mg/mL (Fig. 14D). This trend was only observed at.

the 1 mg/mL concentration (Fig. 14D). By subtracting the absorption signal from the IONPs alone, we applied a correction factor to the data by subtracting the value of the IONP absorption at 0.1 and 0.5 mg/mL. We do not recommend the use of MTT assay at concentrations above 0.5 mg/mL with IONPs unless the IONPs can be completely removed after short-term acute exposures. A two-way ANOVA revealed no statistically significant difference between the measured and corrected values at 0.1 mg/mL. However, statistically significant differences in the corrected and measured values were returned for the 6- and 12-h timepoints p = 0.0349 in both cases (Fig. 15). There was a statistically significant increase in viability at the 12-h timepoint for all both treatment concentrations (0.1 mg/mL and 0.5 mg/mL), compared to measured controls. No statistically significant reduction in viability was observed at any of the time points investigated for the 0.1 mg/mL and 0.5 mg/mL. These results should be interpreted with caution, and additional verification methods, such as qPCR, flow cytometry, and/or clonogenic assays should be used in

combination with this method.

Overall, this investigation showed low, concentration-dependent toxicity of magnetite NPs when coated with alginate. Despite the decrease in viability at the 12-h time point, the cells appear to be recovering by 24 h post exposure. No corresponding increase in cytotoxicity or reduction in metabolic activity was observed to corroborate the decrease in the viability assay at the 12-h timepoint. The measured viability decrease at the 12-h time point may need to be investigated further, as the viability was nearly identical to controls by the 24-h time point. In addition, the cytotoxicity and apoptosis profiles at 12 h do not demonstrate increased toxicity or dead cells. No increase in caspase activity was detected when A549 cells were exposed to IONPs. It appears that the doubling time (growth rate) of the cells may have been altered; possibly halted between 6 and 12 h, then increased again from 12 to 24 h. The low viability measured at 12 h was not observed as apoptosis by this assay; therefore, either cell death had been induced by necrosis, cell proliferation was impaired, or both. Considering the lack of evidence of cytotoxicity of the NPs revealed by the cytotoxicity assay in combination with the low level of observed apoptosis, the main contributing factor to the observation of reduced viability (a low measurement of viable cells) at the 12-h time point is likely attributed to reduced proliferation, both preceded and followed by increased proliferation, as opposed to cell death. Superparamagnetic IONPs can affect cyclin-dependent kinases in human stem cells which may provide a potential mechanism for the promotion of cell proliferation [49]. Therefore, such NPs may very likely have a complex effect on the proliferation cycle in certain human cell lines. This effect and the mechanism(s) thereof merit significant further research.

## 4 Discussion

This study investigated the acute in vitro cytotoxicity of three concentrations of colloidal magnetite NPs, covering a relatively wide range of dosages, in a human lung carcinoma cell line (A549), by comparing mortality, viability, and apoptosis profiles over time. Since the reliability of many of the assays typically used to investigate nanomaterial toxicity has been called into question since many researchers are aware of the potential for fluorescent NPs to enhance the fluorescent signal, or for other metal and metal oxide NPs to absorb the fluorescent signal [50]; 2012; [[51], [52], [53], [54]], we sought to determine the concentration-dependent interference with fluorometric, colorimetric and luminescence assays. We have accounted for the fluorescence signal absorption by the magnetite NPs at the concentrations investigated by incorporating a correction factor based on experimental data. The absorbance data demonstrates that the absorption of visible light is consistent throughout the visible range detected in the assays. Of additional concern are the observed interactions with MTT reagents, apparent at 1 mg/mL, which could not be overcome by introducing an additional correction. The combination of assays has previously been proposed to verify the findings of a single assay in the investigation of NP toxicity [52]; Alinovi et al., 2015), and in our study, the employment of four different assays adds another dimension of quality control to the interpretation of the data.

Previous studies on dextran-coated NPs have shown that detrimental effects of magnetite NPs may be facilitated by the biochemical modifications to dextran by biological systems as well as the weak interaction between the dextran coating and the nanoparticle. Dextran undergoes conformational changes and may completely desorb from the nanoparticle surface (Sonen and De Cuyper, 2010). Cellular uptake of magnetite NPs coated with dextran has been degraded in acidic lysosomes, leaving a rapidly degraded iron core. This iron can then induce toxic reactive oxygen species (ROS) intermediates via the Fenton reaction [55,56]. One of the causes for the weak interaction between dextran and the NP stems from the functional groups binding the hydrocarbon polymer to the metal oxide NP. Dextran uses a hydroxyl (OH^−^) functional group to bind the NP (M^+^). Our coating agent has several OH- carbonyl (COOH^−^) groups emerging from the polymer chain, increasing the negative character, and thus, likely strengthening the bond between the polymer and the NP. This capping method of incorporation of a stronger bonding, FDA-approved polymer is anticipated to reduce ROS-mediated cytotoxicity.

We added IONPs to untreated wells immediately before was the measurement was taken on the plate reader. This additional control could return results on potential interactions between the IONPs and the assay reagents as well as reporting back any optical interference from the signal that should correspond to that of the untreated cells. We ran an entire plate comprising a cell-free system, growth media, assay reagents, or molecular water as blanks [27]. We exposed the luminescence positive control, which was expressing a high luminescence signal, to an IONP concentration curve to determine the level of light absorption as evidenced by signal reduction. Although plate centrifugation or washing prior to measurements to remove NPs [52] has been suggested, due to the length of the time points used in this experiment, we believe it is likely that IONPs may have been taken up by the cells, and we have not accounted for or quantified uptake in this study. Future work may investigate whether monolayers of cells with particles inside have measurable differences in optical properties and determination of measurable concentrations.

Some limitations to this study include the limited range of IONP concentrations investigated (0.1–5 mg/mL), the phase of iron oxide used, and we the capping agent (alginate). Fe_2_O_3_ may produce different results due to the different optical properties, similarly, capping agents may also interact differently with light. Any deviations from these parameters may yield different results, therefore, it is recommended that researchers incorporate these additional control methods into their experiments and add them to standard protocols.

Regarding the relevance of this work to the existing published literature, the data must be interpreted on a case-by-case basis [57]. and colleagues investigated the toxicity of graphite-octadecylamine-CoFe_2_O_4_ in a fibroblast cell line using the MTT assay and report low observed toxicity. However, lacking thorough additional controls, it is possible that they have overestimated the viability as the absorbance may have been artificially increased at the concentrations they investigated (up to 250 μg/mL), similar to our findings at 0.1–0.5 mg/mL. No data on absorbance for these particles is reported in the manuscript, therefore it remains unclear as the degree of interference of this nanocomposite. [58]; investigated biosynthesized zinc ferrite and cobalt ferrite NPs in MCF-7 cells using an MTT assay at low concentrations (30 μg/mL), and although they do not mention additional controls in the assay, according to our data the interference at this NP concentrations is likely to be negligible. Coating materials add an additional complication, as their optical properties and potential for interactions with assay reagents should also be considered. For example, although alginate coating used in this study is generally regarded as having low-toxicity for biomedical applications [59], no data was available on potential interactions of alginate with MTT.

Comprehensive toxicity profiles should include data on toxicity in multiple cell lines in addition to animal models to include investigations on developmental effects. It is important to translate cytotoxic effects revealed by exposure to a concentration in cell culture to a no observed adverse effects level (NOAEL), systemic dose administration, which is not necessarily straightforward. Even in cases where *in vivo* studies have demonstrated a NOAEL, localization in specific organ systems and subsequent toxicity to those specific cell types may not yet have been identified. *In vitro* cytotoxicity of NP systems in specific cell types is also useful for identifying mechanisms of toxicity after systemic toxicity is observed. Also, higher concentrations of the investigational nanomaterial that could be feasibly systemically administered may be investigated in cell culture. This is important for materials that will be targeted to a specific cell type or administered as inhalation aerosols.

## CRediT authorship contribution statement

**Leisha Martin:** Writing – original draft, Visualization, Methodology, Investigation, Formal analysis, Conceptualization. **Kimberly Lopez:** Methodology. **Shayden Fritz:** Methodology, Formal analysis. **Charles P. Easterling:** Visualization, Resources, Methodology. **Jacob A. Krawchuck:** Resources, Methodology, Formal analysis. **Agus R. Poerwoprajitno:** Resources, Methodology, Formal analysis. **Wei Xu:** Writing – review & editing, Supervision, Investigation, Funding acquisition, Conceptualization.

## Declaration of competing interest

The authors declare the following financial interests/personal relationships which may be considered as potential competing interests:

Leisha Martin declares corporate affiliations with MNT SmartSolutions and LEI NanoTech.
